# Corneal thermal burn injuries during long-duration spaceflight: mechanisms, evaluation, and management

**DOI:** 10.1038/s41433-024-03072-7

**Published:** 2024-04-20

**Authors:** Alex Suh, Joshua Ong, Ethan Waisberg, Andrew G. Lee

**Affiliations:** 1 Tulane University School of Medicine, New Orleans, LA USA; 2 Department of Ophthalmology and Visual Sciences, University of Michigan Kellogg Eye Center, Ann Arbor, MI USA; 3 Department of Ophthalmology, University of Cambridge, Cambridge, USA; 4 Center for Space Medicine, Baylor College of Medicine, Houston, TX USA; 5 Department of Ophthalmology, Blanton Eye Institute, Houston Methodist Hospital, Houston, TX USA; 6 The Houston Methodist Research Institute, Houston Methodist Hospital, Houston, TX USA; 7 Departments of Ophthalmology, Neurology, and Neurosurgery, Weill Cornell Medicine, New York, NY USA; 8 Department of Ophthalmology, University of Texas Medical Branch, Galveston, TX USA; 9 University of Texas MD Anderson Cancer Center, Houston, TX USA; 10 Texas A&M College of Medicine, Bryan, TX USA; 11 Department of Ophthalmology, The University of Iowa Hospitals and Clinics, Iowa City, IA USA

**Keywords:** Education, Risk factors

Embarking on long-duration spaceflights with limited resources and absence of immediate medical facilities necessitates careful consideration of potential health hazards in the unforgiving confines of space. Among the extensive possible risks inherent in space exploration, ocular injuries [1–3], particularly corneal thermal burns, stand out as significant focal points of concern. The Lifetime Surveillance of Astronaut Health database highlights numerous ocular injuries in the history of the U.S. space program, prompting concerns about potential vision-threatening injuries on the International Space Station (ISS) [4]. Reports of ocular, orbital, and cranial trauma during various ISS missions exist, but as of now, none have yet been secondary to a corneal thermal burn [5]. Recognizing the critical importance of understanding and efficiently addressing these injuries in space, this paper explores the unique circumstances that make corneal thermal burns a critical health concern for the Artemis Program and Martian missions. As the scope and duration of space exploration expands, especially in the context of commercialization, proactive approaches towards developing comprehensive protocols must addressed. This paper aims to discuss onboard diagnostic tools and potential modalities for treating thermal burns of the cornea during spaceflight with the current medications available on the ISS.

## Risk Factors for Corneal Thermal Burns During Spaceflight

Examining the equipment used on the ISS reveals potential hazards and exposures to corneal thermal burns. Tools like the space oven for meal preparation highlight sources of heat the crewmembers interact with daily during their missions. The Zero G Kitchen Space Oven utilizes electrical heating elements to heat food to a nominal temperature of 177 degrees Celsius (350 degrees Fahrenheit) [6]. Although there is a cooling rack, and the space oven is thoroughly tested before integration into the ISS, technical malfunctions or exposure to hot food particles may induce a corneal burn. Moreover, the limited and confined nature of the spacecraft amplifies the risk of thermal burns, as the proximity of equipment and limited spatial freedom heightens the likelihood of accidental exposure to sources of heat. Crewmembers are also notably susceptible to radiant energy burns (aka flash burns) from direct or reflective UV light from the sun [7].

During a Apollo-Soyuz test project in 1975, propellant and combustion products (e.g., hydrazine, nitrogen oxide, nitrogen tetroxide) were inadvertently admitted into the cabin during re-entry [8]. In a hypothetical scenario with similar conditions aboard the ISS, the propellants could combine with other reagents to create an exothermic reaction and a potential burn risk to crewmembers [4]. Scientific experiments with flames or exothermic chemical reactions may put crewmembers at increased risk of obtaining corneal thermal burns as well. Terrestrially, gravity draws colder, denser air to the base of flames, thereby displacing hotter, lighter air upward. In a microgravity environment, flames do not exhibit upward flow and thus produce more unpredictable behaviours for combustion [9]. Flames in microgravity also may survive in less oxygen environments and burn for longer periods of time, thus increasing risk of thermal burns if an uncontrolled fire were to ignite [10].

## Diagnostic and Medical Management

For crewed missions without a medical doctor, a designated medical officer is appointed who undergoes 40 h of paramedic-level training. This comprehensive training includes skills such as administering injections, suturing wounds, and implementing eye-washing protocols [11]. In the event of a corneal thermal burn injury, management should be initiated immediately with copious irrigation. A Space Eye-Wash apparatus is available aboard the ISS, complete with a saline solution for continuous irrigation. The device aids in removing potential foreign bodies and chemical exposures [12]. Topical anaesthetic (tetracaine (0.5%, 15 mL) is also available [13]. Following the eye-wash, the medical officer can complete the ocular examination including topical fluorescein staining of the cornea [14]. The crew also has access to remote health guidance from the terrestrial based flight surgeon and other subject matter experts on the ground at NASA’s Johnson Space Center. Remote imaging including ocular photography, an onboard portable ocular ultrasound, and an optical coherence tomography (Heidelberg Spectralis OCT2) are available on ISS [15].

The blink reflex typically prompts the eye to close when exposed to a stimulus taking longer than 0.1 s to travel to the eye [16]. Consequently, terrestrial thermal burns often impact the eyelid more than the conjunctiva or cornea. Mild eyelid burns can be irrigated with sterile isotonic saline solution, followed by the application of an ophthalmic antimicrobial ointment [17]. Fortunately, the risks for thermal burn in space impacting the conjunctiva or cornea is low and most mild cases resolve without significant complications [18]. Treatments available on the ISS include oral anti-inflammatory and analgesic treatments (Acetaminophen (Tylenol) 325 mg, Aspirin 325 mg, Ibuprofen (Motrin) 400 mg, Ketorolac (Toradol) 30 mg/mL (2 mL)); topical cycloplegic mydriatics (e.g., cyclopentolate (Cyclogyl, 2%, 15 mL) and tropicamide (Mydriacyl, 1%, 15 mL)); and topical ophthalmic antibiotics (Erythromycin ointment (0.5%); Moxifloxacin (Vigamox, 0.5%); Tobramycin and Dexamethasone (Tobradex), 0.3%; 0.1%) [13]. Cool saline compresses and adequate lubrication with artificial tears (Refresh Plus, 0.5%); Hypromellose (Nature’s Tears, 0.4%); Mineral Oil and White Petrolatum (Refresh PM, 42.5%; 57.3%, 3.5 gm) can also be given as comfort measures.

In contrast to ISS missions, where evacuation to Earth is possible with a Crew Return Vehicle, lunar sorties, lunar outposts, Near-Earth missions, and a Mars mission do not provide evacuation options for an urgent return trip to Earth [19]. Additionally, the communication latencies, with up to 20 min for the signal to travel to and from Mars, will add complexity to medical decision-making processes [20]. Given the rapid advancements in machine learning, the integration of artificial intelligence (AI) into telemedicine may prove beneficial for determining optimal management steps, particularly in cases of corneal thermal burns where communication delays impede immediate intervention during medical emergencies [21]. Large language models specifically trained for space medical emergencies can potentially provide a suitable response in the absence of a doctor [22–24].

## Conclusion

Although the risk for corneal thermal burn during spaceflight is low, awareness of the risks and prevention (e.g., safety goggles) may mitigate the need for potentially mission threatening interventions. Onboard ocular imaging technology supported by remote guidance on the Earth can manage most minor (e.g., corneal abrasion) or mild (eyelid thermal exposure) injuries. More severe injuries pose a greater risk in austere and distant environments (e.g., the moon or Mars) and AI enabled autonomous or semi-autonomous telemedicine may be useful countermeasures especially given the potential communication signal delay created by long distance space exploration. Testing these risk protocols in advance including virtual reality and computerized simulation may be warranted to reduce the risk for future long duration space travel.

## References

[1] Waisberg E, Ong J, Masalkhi M, Lee AG, Berdahl J. Anatomical considerations for reducing ocular emergencies during spaceflight. *Ir J Med Sci 1971* -. Published online May 27, 2023. 10.1007/s11845-023-03407-5. 10.1007/s11845-023-03407-5 PMC10808690 37243845

[2] Ong J, Waisberg E, Masalkhi M, Kamran SA, Lowry K, Sarker P, et al. Artificial Intelligence Frameworks to Detect and Investigate the Pathophysiology of Spaceflight Associated Neuro-Ocular Syndrome (SANS). Brain Sci. 2023;13:1148 10.3390/brainsci13081148 37626504 10.3390/brainsci13081148 PMC10452366

[3] Waisberg E, Ong J, Masalkhi M, Zaman N, Kamran SA, Sarker P, et al. The Case for Expanding Visual Assessments During Spaceflight. *Prehospital Disaster Med*. Published online June 27, 2023:1-4. 10.1017/S1049023X23005964. 10.1017/S1049023X23005964 PMC10445111 37365808

[4] Meer E, Grob S, Antonsen EL, Sawyer A. Ocular conditions and injuries, detection and management in spaceflight. NPJ Microgravity. 2023;9:37 10.1038/s41526-023-00279-y 37193709 10.1038/s41526-023-00279-y PMC10188687

[5] Meer E, Grob SR, Lehnhardt K, Sawyer A. Ocular complaints and diagnoses in spaceflight. Npj Microgravity. 2024;10:1–7. 10.1038/s41526-023-00335-7 38167407 10.1038/s41526-023-00335-7 PMC10762130

[6] Space Oven. Zero G Kitchen. Accessed February 5, 2024. https://www.zerogk.space/space-oven

[7] Waisberg E, Ong J, Paladugu P, Kamran SA, Zaman N, Tavakkoli A, et al. Radiation-induced ophthalmic risks of long duration spaceflight: Current investigations and interventions. *Eur J Ophthalmol*. Published online December 27, 2023:11206721231221584. 10.1177/11206721231221584 10.1177/11206721231221584 38151034

[8] Johnson GW Gary Johnson: Lessons Learned from 50+ Years in Human Spaceflight and Safety. Published online April 30, 2018. Accessed January 5, 2024. https://ntrs.nasa.gov/citations/20190028301

[9] Fire in Microgravity. American Scientist. Published February 6, 2017. Accessed February 5, 2024. https://www.americanscientist.org/article/fire-in-microgravity

[10] Magazine S In Space, Flames Behave in Ways Nobody Thought Possible. Smithsonian Magazine. Accessed February 5, 2024. https://www.smithsonianmag.com/science-nature/in-space-flames-behave-in-ways-nobody-thought-possible-132637810/

[11] Medical Operations Team Activities. JAXA Human Spaceflight Technology Directorate. Accessed January 18, 2024. https://humans-in-space.jaxa.jp/en/biz-lab/med-in-space/healthcare/medops/system/

[12] Ocular Burns and Chemical Injuries: Background, Pathophysiology, Etiology. Published online September 18, 2023. Accessed November 4, 2023. https://emedicine.medscape.com/article/798696-overview

[13] NASA. Medical Kit - Contents and Reference. Published online April 9, 2015. chrome-extension://efaidnbmnnnibpcajpcglclefindmkaj/ https://www.nasa.gov/wp-content/uploads/2015/03/medical_kit_checklist_-_full_release.pdf

[14] Bawany S, Macintosh T, Ganti L. Ocular Thermal Burn Injury in the Emergency Department. Cureus. 2020;12:e7137 10.7759/cureus.7137 32257682 10.7759/cureus.7137 PMC7105268

[15] Sargsyan AE, Dulchavsky AG, Adams J, Melton S, Hamilton DR, Dulchavsky SA. Ultrasound detection of simulated intra-ocular foreign bodies by minimally trained personnel. Aviat Space Environ Med. 2008;79:58–61. 10.3357/asem.2191.2008 18225781 10.3357/asem.2191.2008

[16] Waisberg E, Ong J, Masalkhi M, Memon H, Lee AG. Cheers not tears: champagne corks and eye injury. *BMJ*. Published online December 20, 2023:p2520. 10.1136/bmj.p2520 10.1136/bmj.p2520 38123192

[17] Ocular Burns - Injuries; Poisoning. Merck Manuals Professional Edition. Accessed February 5, 2024. https://www.merckmanuals.com/professional/injuries-poisoning/eye-trauma/ocular-burns

[18] Fitzgerald O’Connor E, Frew Q, Din A, Pleat J, Ashraff S, Ghazi-Nouri S, et al. Periorbital burns – a 6 year review of management and outcome. Burns J Int Soc Burn Inj. 2015;41:616–23. 10.1016/j.burns.2014.08.022 10.1016/j.burns.2014.08.022 25406883

[19] HumanResearchWiki. Eye Penetration (Foreign Body). https://humanresearchroadmap.nasa.gov/Evidence/medicalConditions/Eye_Penetration_(Foreign_Body).pdf

[20] mars.nasa.gov. Communications - NASA. Accessed January 18, 2024. https://mars.nasa.gov/mars2020/spacecraft/rover/communications/

[21] Ahn H. Artificial intelligence method to classify ophthalmic emergency severity based on symptoms: a validation study. BMJ Open. 2020;10:e037161 10.1136/bmjopen-2020-037161 32624476 10.1136/bmjopen-2020-037161 PMC7337880

[22] Waisberg E, Ong J, Masalkhi M, Zaman N, Kamran SA, Sarker P, et al. Generative pre-trained transformers (GPT) and space health: a potential frontier in astronaut health during exploration missions. Prehosp Disaster Med. 2023;38:1–5. 10.1017/S1049023X23005848. Published online June 2 10.1017/S1049023X23005848 PMC10445113 37264946

[23] Alser M, Waisberg E. Concerns with the usage of ChatGPT in Academia and Medicine: a viewpoint. Am J Med Open. 2023;9:100036 10.1016/j.ajmo.2023.100036. 39035060 10.1016/j.ajmo.2023.100036 PMC11256230

[24] Paladugu PS, Ong J, Nelson N, Kamran SA, Waisberg E, Zaman N, et al. Generative Adversarial Networks in Medicine: Important Considerations for this Emerging Innovation in Artificial Intelligence. *Ann Biomed Eng*. Published online July 24, 2023. 10.1007/s10439-023-03304-z 10.1007/s10439-023-03304-z 37488468

